# RNA-Sequencing Identification of Genes Supporting HepG2 as a Model Cell Line for Hepatocellular Carcinoma or Hepatocytes

**DOI:** 10.3390/genes15111460

**Published:** 2024-11-13

**Authors:** Paula Štancl, Paula Gršković, Sara Držaić, Ana Vičić, Rosa Karlić, Petra Korać

**Affiliations:** 1 Bioinformatics Group, Division of Molecular Biology, Department of Biology, Faculty of Science, University of Zagreb, 10000 Zagreb, Croatia; pstancl@bioinfo.hr (P.Š.); sara.drzaic@student.pmf.hr (S.D.); 2 Biomedical Research Group, Division of Molecular Biology, Department of Biology, Faculty of Science, University of Zagreb, 10000 Zagreb, Croatia; paula.grskovic@biol.pmf.hr; 3 Department of Obstetrics and Gynecology, Clinical Hospital “Sveti Duh”, 10000 Zagreb, Croatia; vicic.ana@gmail.com

**Keywords:** transcriptomics, HepG2, hepatocellular carcinoma, primary hepatocytes

## Abstract

**Background/Objectives:** Cell lines do not faithfully replicate the authentic transcriptomic condition of the disease under study. The HepG2 cell line is widely used for studying hepatocellular carcinoma (HCC), but not all biological processes and genes exhibit congruent expression patterns between cell lines and the actual disease. The objective of this study is to perform a comparative transcriptomic analysis of the HepG2 cell line, HCC, and primary hepatocytes (PH) in order to identify genes suitable for research in HepG2 as a model for PH or HCC research. **Methods**: We conducted a differential expression analysis between publicly available data from HCC patients, PH, and HepG2. We examined specific overlaps of differentially expressed genes (DEGs) in a pairwise manner between groups in order to obtain a valuable gene list for studying HCC or PH using different parameter filtering. We looked into the function and druggability of these genes. **Conclusions**: In total, we identified 397 genes for HepG2 as a valuable HCC model and 421 genes for HepG2 as a valuable PH model, and with more stringent criteria, we derived a smaller list of 40 and 21 genes, respectively. The majority of genes identified as a valuable set for the HCC model are involved in DNA repair and protein degradation mechanisms. This research aims to provide detailed guidance on gene selection for studying diseases like hepatocellular carcinoma, primary hepatocytes, or others using cell lines.

## 1. Introduction

Hepatocellular carcinoma (HCC) is the most common type of primary liver cancer and the second most common cause of cancer mortality worldwide [1]. Generally, HCC predominates in men and older populations, though its incidence varies by geographic region, age, sex, and race/ethnicity due to differing risk factors [2]. Risk factors include HBV/HCV infection, alcohol consumption, metabolic syndrome, diabetes, obesity, non-alcoholic fatty liver disease (NAFLD), aflatoxin B1 exposure, tobacco use, and family history of HCC [3]. Over one million people are projected to be diagnosed with liver cancer by 2025 [3], highlighting the need for further research and improved treatments.

HepG2 is the most commonly used cell line model for HCC, as indicated by the number of studies in the NCBI database [4]. HepG2 was originally isolated from what was believed to be HCC in a 15-year-old Caucasian male [5], but Lopez-Terrada et al. found that HepG2 shares more characteristics with hepatoblastoma (HB) than with HCC, such as the loss of chromosome 4q [3], trisomies 2 and 20 [6], and a large deletion in exon 3 of the β-catenin (CTNNB1) gene [6,7].

HepG2 is also used as a model for primary hepatocytes (PH) in studies on the toxic effects of heavy metals, nanoparticles, and drugs in vitro [8,9,10]. However, HepG2 cells have reduced or absent expression of the cytochrome P450 (CYP) superfamily, which is crucial for liver metabolic processes [11]. This reduction in cytochrome P450 expression may lead to an underestimation of the toxicity of chemicals metabolized by these enzymes in PH. Despite this, HepG2 can be used as a model for phase II enzymes since their expression is similar in both PH and HepG2 [12].

The HepG2 transcriptome has been compared with other hepatic cell lines [13], HepG2 cells under different experimental conditions [14], and liver cells [13,14,15] to evaluate its suitability as a model cell line. While the study by Arzumanian et al. showed significant gene expression differences between HepG2 and liver cells [13], other studies showed that the gene expression is similar between HepG2 and liver cells, but with a few exceptions, which have to be taken into account when choosing an appropriate cell model [14,15]. This study aims to further emphasize differences in gene expression between HepG2, PH, and HCC. Identifying differentially expressed genes can help assess HepG2’s suitability as a model for research focused on PH or HCC.

## 2. Materials and Methods

### 2.1. Publicly Available Datasets

Publicly available datasets used in this study are available in the Gene Expression Omnibus (GEO) database under accession codes GSE88089 and GSE35585 for HepG2, GSE43984 and ERR030887 in Sequence Read Archive for PH, and GSE105130 for normal and HCC tissues from 25 patients. Information about samples is available in Supplementary Table S1.

### 2.2. Sequencing of HepG2 Cell Line

Dulbecco’s modified Eagle’s medium (DMEM, Lonza, Basel, Switzerland) supplemented with 10% (*v/v*) fetal bovine serum (FBS) (Gibco, Billings, MT, USA), 4 mM L-glutamine (Lonza), 100 U/mL penicillin, and 100 μg/mL streptomycin (Lonza) was used for growing HepG2 cells with 5% CO_2_ at 37 °C. RNA was isolated using a QIAGEN RNeasy Micro kit (Qiagen, Hilden, Germany) according to the manufacturer’s protocol and sent to the Genomics Core Facility at EMBL for RNA sequencing.

### 2.3. Karyotype Coordinates

Program CytoConverter (Cytogenetic Nomenclature to Genomic Coordinate Translator) [16] was used to obtain the genomic coordinates of reference and in-house HepG2 cell line rearrangements from its karyotype. Our in-house HepG2 cell line karyotype is 51(48–52)<2n>XY, +2, −14, +20, +11p, +t(16;?), +t(17q;6p), +mar,t(1;21)(p22.2;p11–12). The reference HepG2 cell line karyotype we used is 52(47–54)<2n>XY, +2, +14, +17, +20, +2mar, t(1;21) (p22.2;p11–12), i(17q)/der(17)t(17;17)(p11;q11) [17]. Karyotype coordinates are listed in Supplementary Table S2.

### 2.4. Quality Control and Mapping

Quality control of downloaded raw reads was assessed by FASTQC (version v0.11.9) [18]. Raw reads were trimmed using a sliding window of 10 and a quality score below 25, adapters were removed and reads shorter than 60 bp and average quality below 20 were filtered by Trimmomatic (version 0.32) [19]. Reads were mapped onto the human genome (hg38) using STAR (version 2.5.3a) [20] with the default settings. Mapped reads were counted over gene features annotated from Ensembl (GRCh38.105) using featureCounts v.2.0.0. [21].

### 2.5. Differential Expression Analysis

Genes with less than 10 reads across all samples were filtered out to reduce noise, improve statistical power, and ensure more reliable results in downstream differential expression analysis. Raw counts were transformed using variance stabilizing transformation. Transformed reads were used for principal components analysis and average clustering of samples based on Euclidean distance using the pheatmap function from the R pheatmap package (version 1.0.12) [22]. The DESeq2 package (version 1.42.0) [23] in R was used to compute statistical fold changes using LFC shrinkage from R package ashr (version 2.2.66) [24] in gene expression of protein-coding genes. The results of differential expression analyses between all groups were visualized with MA plots. Differentially expressed genes (DEGs) were considered to be statistically differentially expressed, either upregulated or downregulated, if their adjusted *p* values were smaller than 0.001 and absolute values of log fold change higher than 1.33. The UpsetR plot [25] was constructed based on the overlapping differentially expressed genes between different pairwise comparisons. The Chi-square test was used to compare DEGs in HepG2-specific regions to HepG2 karyotypes. Transcripts Per Million (TPM) were calculated from raw counts and used for visual comparison of genes belonging to the CYP and IGB family, as well as genes considered to be an important biomarker for HCC. We looked at the list of identified DEGs overlapping between two different sets of parameters (adjusted *p*-value of 0.001 and log fold change of 2, and an adjusted *p*-value of 0.05 and log fold change of 1.33) to look only at genes appearing in stricter filtering conditions. We examined the DEGs found between HCC and PH, which were also found between HepG2 and PH, representing a set of genes valuable to studying HCC. Also, a list of valuable genes to study the PH was obtained by examining DEGs found between HCC and PH, which were also found between HepG2 and HCC to study PH.

### 2.6. Functional Analysis

Gene ontology (GO) annotation and Kyoto Encyclopedia of Genes and Genomes (KEGG) pathway analysis were performed using an over-representation analysis (ORA) approach from the clusterProfiler R package [26]. GO annotation consists of three parts: biological process (BP), cellular component (CC), and molecular function (MF). The DEGs were organized into upregulated (log2FC  >  0) lists and downregulated (log2FC  <  0) lists. Gene enrichment analysis was performed separately on each list. Significantly enriched gene sets were considered with Benjamini–Hochberg adjusted *p*-value  <  0.05.

### 2.7. Drug–Gene Interaction Database

Gene–drug interactions were identified using the R package rDGIdb [27,28], which provides information about the druggability of genes. This analysis focused on genes identified as relevant for HCC and primary hepatocyte models in HepG2. The number of drugs interacting with each gene was quantified to assess the potential impact of druggable genes on research findings in both HCC and PH models.

### 2.8. Statistics and Reproducibility

All statistical analyses and visualizations were performed in the R software (version 4.3.3) environment [29]. Fisher’s exact test and Chi-square test were used to test whether a statistically significant relationship exists between two proportions. The DESeq2 package internally uses a two-sided Wald test. *p* values were corrected for multiple comparisons using the Benjamini–Hochberg procedure.

## 3. Results

### 3.1. Identification of Differentially Expressed Genes (DEGs) Between HCC, HepG2 Cells, and PH

**Figure 1:**
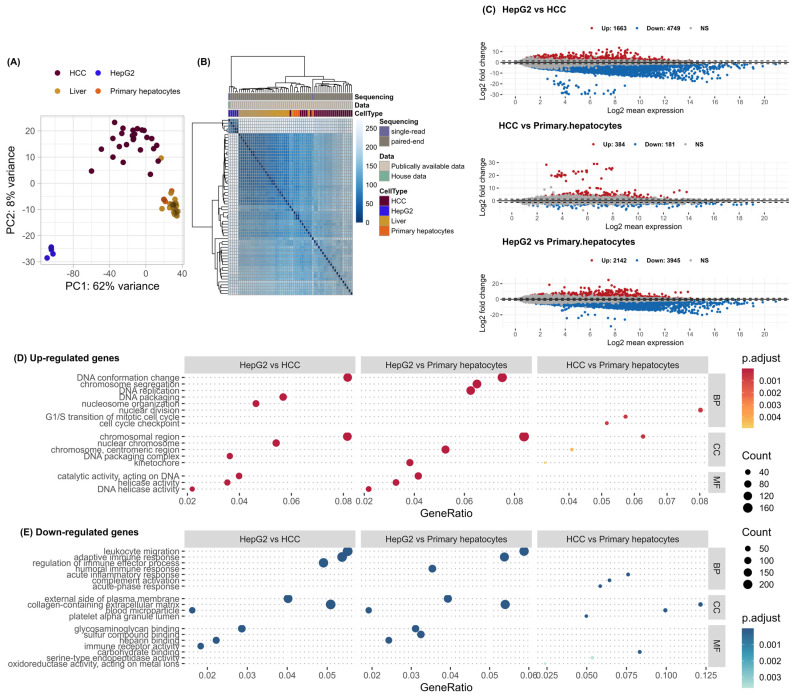
(**A**) Principal component analysis; (**B**) unsupervised clustering using complete clustering of Euclidean distance; (**C**) MA plots showing differentially expressed genes between three subgroups; (**D**) over-representational analysis of upregulated genes and downregulated genes (**E**) between groups.

Principal component analysis (PCA) and unsupervised clustering using Euclidean distance were used to assess the similarity of gene expression levels between 25 HCC patients and their adjacent normal liver tissue, five HepG2, and four PH cell lines. The first principal component (PC1) accounts for 62% of the overall variance of the dataset, while the second principal component (PC2) accounts for 8% (Figure 1A). Unsupervised clustering showed that the samples were grouped according to their type (HCC, HepG2, PH, and adjacent non-tumor liver tissue from HCC patients) (Figure 1B). Based on differential expression analysis of pairwise comparison of different groups (Supplementary Table S3), we identified 1663 up- and 4749 downregulated genes in HepG2 compared to HCC as well as 2142 upregulated and 3945 downregulated genes in HepG2 compared to PH. The lowest number of DEGs was observed between HCC and PH, with only 384 up- and 181 downregulated genes (Figure 1C). Upregulated and downregulated DEGs were annotated separately based on three GO categories: biological processes (BP), cellular compartments (CC), and molecular functions (MF) (Figure 1D,E). The terms most commonly associated with the upregulated genes in HepG2 compared to HCC or PH were involved in chromosome organization, DNA repair and replication mechanisms, and cell cycle. Similar pathways were also dominantly represented among the upregulated genes in HCC compared to PH but with fewer DEGs compared to the HepG2 vs. HCC and HepG2 vs. PH subgroups (Figure 1D, Supplementary Table S4). Most downregulated DEGs between subgroups are associated with various immunological terms such as adaptive and humoral immune response, cell migration, blood coagulation, and lipid and steroid metabolic processes (Figure 1E, Supplementary Table S4). We observed that the xenobiotic metabolic process (GO:0006805) and various drug metabolic processes were downregulated only in HepG2 compared to HCC and PH (Supplementary Table S4). As for KEGG annotation, the upregulated genes in the HepG2 compared to HCC and PH were involved in viral carcinogenesis, neutrophil extracellular trap formation, alcoholism, transcriptional dysregulation in cancer, necroptosis, and cell cycle (Supplementary Table S5a). KEGG pathways analysis of downregulated genes in the HepG2 vs. HCC or PH showed they were involved in retinol and drug metabolism by cytochrome P450 and other enzymes, cytokine–cytokine receptor interaction, phagosome, and steroid hormone biosynthesis (Supplementary Table S5b). No significant KEGG pathways involving the DEGs in the HCC vs. PH subgroup were detected.

We also made pairwise comparisons of adjacent non-tumor liver tissue from HCC patients to the other three groups (HepG2, HCC, and PH) (Supplementary Table S6). The highest number of 7464 DEGs was detected in the comparison of non-tumor liver tissue with HepG2, followed by 1881 DEGs in comparison with HCC and only 132 DEGs in comparison with PH (Supplementary Figure S1). The majority of DEGs, which were downregulated in non-tumor liver tissue compared to HCC and HepG2, are involved in DNA replication and structure. On the other hand, DEGs, which were downregulated in non-tumor liver tissue compared to PH, are involved in unfolded protein response. In the downstream analysis, we only focused on pairwise comparisons between HepG2, HCC, and PH in order to investigate the DEGs between these groups of comparisons.

### 3.2. HepG2 Karyotype Regions Are Not Enriched with DEGs

**Figure 2:**
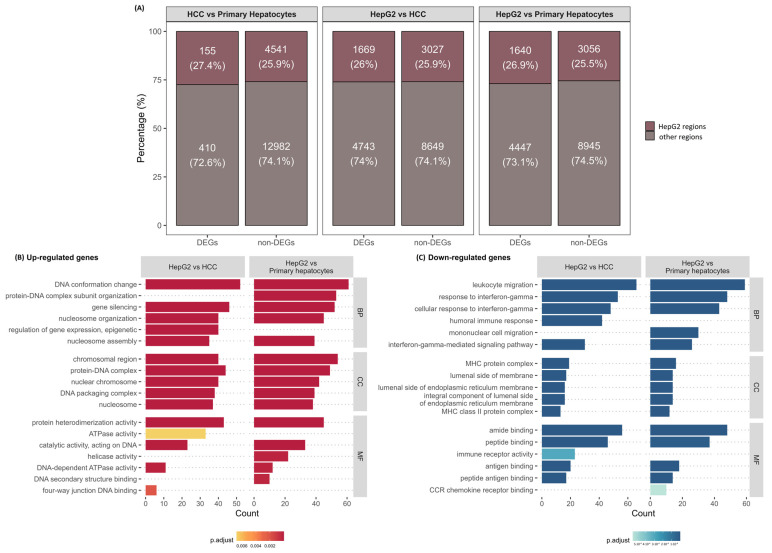
(**A**) Percentage of differentially expressed genes (DEGs) located in in-house HepG2-specific karyotype regions and the other regions (Chi-square test, *p* > 0.05) between different pairwise comparisons of HepG2, HCC, and primary hepatocytes (PH); (**B**) over-representational analysis showing 5 significantly over-represented terms in biological processes, cellular compartmentalization and molecular function of upregulated genes and downregulated genes in HepG2 karyotype regions (**C**) between groups HepG2 and HCC, as well as HepG2 and PH.

We proceeded to compare the fractions of DEGs in genome structural regions specific to the HepG2 karyotype from the sequenced in-house HepG2 cell line and a reference HepG2 cell line. When overlapping all identified DEGs with HepG2-specific regions from the karyotype in-house sequenced HepG2 cell line and the rest of the karyotype, no statistically significant difference in DEGs localization was observed in any of the pairwise comparisons (Fisher’s exact test, *p*-value > 0.05) (Figure 2A). Overrepresentation analysis of upregulated DEGs found in the HepG2-specific karyotype regions showed they were associated with DNA conformation change and organization as biological functions, as well as with chromosomal regions as cellular compartments (Figure 2B). On the other side, downregulated DEGs in the HepG2-specific regions were mostly immune-related GO terms in HepG2 compared to HCC or PH and with organic acid catabolic metabolism and xenobiotic transport (Figure 2B). Identical results were obtained by analyzing HepG2-specific regions from the reference karyotype HepG2 cell line (Supplementary Figure S2).

### 3.3. Differentially Expressed 187 Genes Between PH, HepG2, and HCC Show Distinct Clusters of Various Functions

**Figure 3:**
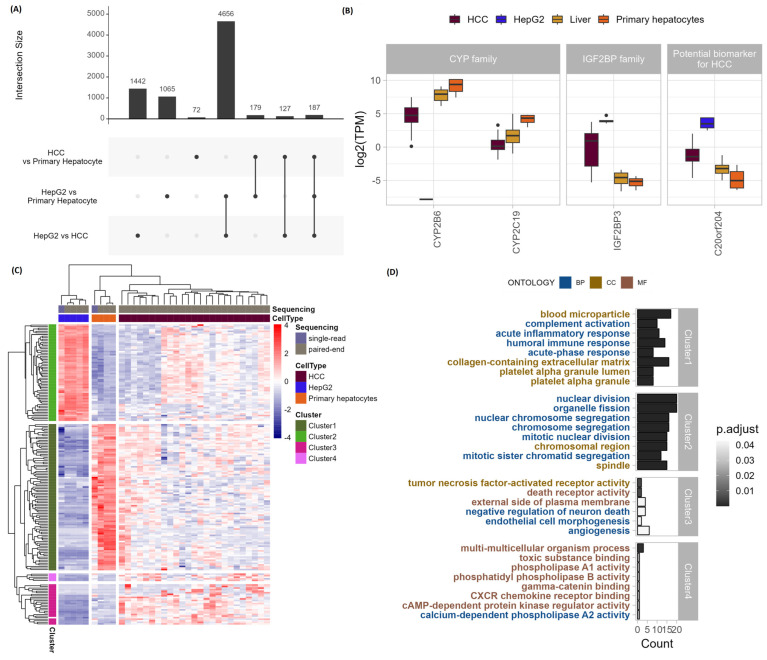
(**A**) Upset plot of differentially expressed genes across different pairwise comparison groups of differential expression analysis of HepG2, PH, and HCC. (**B**) Transcription per million (TPM) of DEGs found to be differentially expressed in all 3 pairwise comparisons that play an important part in differentiating HCC from HepG2 and PH. (**C**) Heatmap of expression of 187 DEGs between all pairwise comparisons. (**D**) Overrepresentation analysis of 187 DEGs between all pairwise comparisons in identified clusters in (**C**).

To gain insight into different DEGs between different groups, we examined the overlaps of DEGs between HCC and PH, HepG2 and PH, and HepG2 and HCC. The overlaps are visualized with an Upset plot shown in Figure 3A. The highest number of overlapping DEGs, 4656 in total, appeared in a pairwise comparison of the HepG2 vs. HCC subgroup with the HepG2 vs. PH subgroup, indicating their expression in HCC and PH was similar. Most of the total 1442 genes whose expression differed only between HepG2 and HCC were upregulated in biological functions such as peptide biosynthetic process, molecular functions associated with RNA binding, and catalytic activity acting on RNA (Supplementary Table S7a). Upregulated DEGs unique for the HepG2 vs. PH subgroup were mostly involved in DNA metabolic and cell-cycle processes, while downregulated DEGs were involved in small molecule catabolic, mitochondrial localized, and alcohol biosynthetic processes (Supplementary Table S7b). The smallest number of DEGs was observed exclusively between HCC and PH.

Genes identified as differentially expressed between all groups include important genes of the CYP family, which are not expressed or have low expression in the HepG2 cell line, as well as IG2BP families, which have roles in tumorigenesis and tumor progression (Figure 3B). In this group, we also detected gene C20orf204, which encodes an HCC-specific protein promoting cell proliferation. The identified 187 DEGs between all subgroups cluster into four distinct clusters, shown in Figure 3C. Cluster 1 contains enriched gene ontology (GO) terms involved in the ECM and response to stimulus and stress, all of which are higher in PH and HCC compared to HepG2. Processes involved in division and chromosome organization are characteristic of cluster 2, where the genes are most expressed in HepG2, followed by the expression in HCC. Genes associated with death receptor activity characterize cluster 3, which has the highest expression in HCC patients compared to both cell lines (Figure 3C,D).

### 3.4. Defining Potential Genes for Studying HepG2 as a Representative Model for Primary Hepatocytes and HCC

In order to identify genes that might indicate the usage of HepG2 as a good model for studying HCC and PH, we searched for differentially expressed genes overlapping between different pairwise comparison groups. DEGs between HCC and PH that also overlap with DEGs between HepG2 and HCC could be a valuable gene set whose expression is similar in HepG2 and PH, making HepG2 a good model for PH in the experiments researching the products of these genes (Supplementary Table S8a). Likewise, DEGs identified between HepG2 and PH that also overlap with DEGs between HCC and PH could be researched in HepG2 as a good model for HCC (Supplementary Table S8b). When examining different filtering options and stringent and relaxed parameters, we identified 21 out of 421 and 40 out of 397 genes for HepG2 as valuable models for PH and HCC, respectively (Table 1).

The greatest number of genes indicating HepG2 is a good model for PH is involved in the transmembrane transfer of molecules/ions/electrons (6) and transcription regulation (3), while two genes play a part in cytoskeleton regulation and neuronal communication, respectively. MAGEC2, an enhancer of ubiquitin ligase TRIM28, which regulates p53 degradation, is the only gene from this group with an association with HCC development and was shown to be overexpressed in HCC compared to PH and HepG2.

On the contrary, most genes representing HepG2 as a good model for HCC are involved in mitotic spindle regulation (6), immune response (6), protein degradation (5), molecule/ion transfer (4), and DNA repair (4). Unlike genes between HepG2 and PH, more genes with a similar expression between HepG2 and HCC are involved in translation regulation than in transcription regulation, DNA repair, protein degradation, and immune system response. Three out of 41 genes play a part in cytoskeleton regulation, two genes influence neuronal communication, and two genes are part of metabolic pathways. Multiple gene products that we detected to be useful in HepG2 investigation of HCC have been shown to have a role in the liver or HCC development: a translation regulator TRIM71, UBE2T involved in lipid metabolism and actin regulation, GNAZ, which is a G-protein mediator of signaling pathways. Moreover, we also identified RAB11FIP4, which enhances the metastatic potential of HCC in hypoxic conditions; OIT3, which is involved in liver development; HKDC1, a member of the hexokinase protein family involved in glucose homeostasis and hepatic lipid accumulation; and SERPINA1, an inhibitor of serine proteases associated with chronic liver disease.

Other genes, such as potent oncogenes AFP and ACSL4, as well as tumor suppressor HAMP, which is also associated with HCC development, can be found in a broader list of 397 genes for HepG2 as a valuable model for HCC.

**Figure 4:**
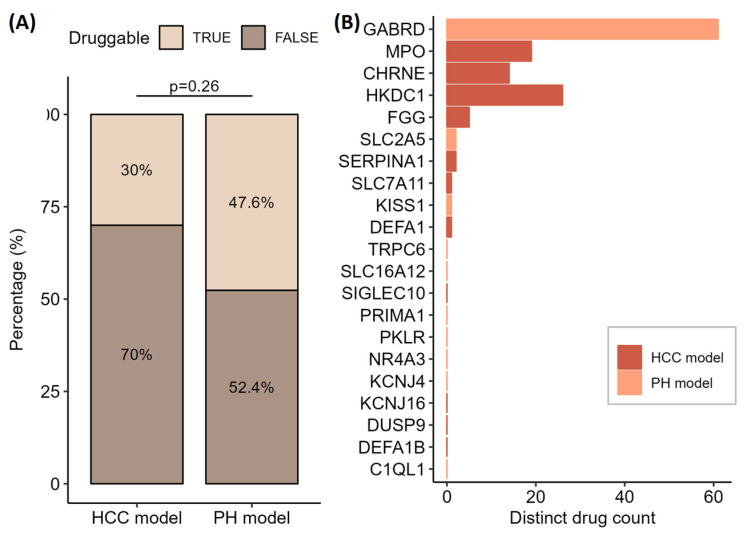
(**A**) Percentage of genome druggable genes from a valuable list of 21 and 40 genes for hepatocellular carcinoma (HCC) and primary hepatocytes model, respectively. There is not a significant difference in the enrichment of druggable genes detected between the HCC and PH model list of genes (Fisher’s exact test, *p* = 0.26). (**B**) Number of distinct drugs for each of the druggable genes in the HCC and PH model list.

For all 61 genes, we looked at their druggability in the drug–gene interaction database (Figure 4A) to see which ones can be therapeutically modulated by medicines. A larger proportion of druggable genes were found in the gene list of the primary hepatocytes model (Fisher’s exact test, *p* = 0.26). Despite that, more druggable genes with at least one or more distinct drugs were found in the HCC model (Figure 4B). When examining the complete dataset of 397 genes for the HCC model and 421 genes for the PH model, we found 144 and 148, respectively, druggable genes (Fisher’s exact test, *p* = 0.77).

## 4. Discussion

The HepG2 cell line is a valuable model for a wide range of studies involved in drug metabolism and hepatotoxicity studies [9,30], and it is also a model for hepatocellular carcinoma and hepatocytes [31,32]. Our research, along with findings from other studies [12,33], has found lower or no expression of genes involved in drug metabolism, questioning the validity of using HepG2 for these kinds of studies, which are still quite frequent in vitro. When examining specific regions of the HepG2 karyotype, we did not observe significant differences in the enrichment of DEGs compared to other regions. However, the functional processes associated with the genes found in the HepG2-specific karyotype regions align with the overall differences we observed when analyzing all differentially expressed genes between the groups. This suggests that the observed gene expression patterns in the HepG2-specific karyotype regions reflect the broader differences observed in our study.

One of the genes found to be a valuable resource for investigating PH, but not HCC, using the HepG2 cell line is *MAGEC2*. Riener et al. first identified *MAGEC2* as one of the genes whose protein product expression is frequently increased in HCC [34]. An increased expression of *MAGEC2* was afterward shown to be an unfavorable factor in overall survival for patients diagnosed with HCC [35]. The same study showed that the expression of this gene in HCC did not correspond to its expression in the HepG2 cell line, which matches our findings that its expression has similar levels in HepG2 and PH but differs from its expression in HCC. In more relaxed filtering, we also found other genes that have important implications in HCC similar to *MAGEC2*, such as *ZIC2*, *PTHLH*, *EEF1A2*, and *RGCC*, which are similarly expressed in HepG2 and PH, but differently expressed in HCC [36,37,38,39]. Moreover, we found that HepG2 is a good model for PH in research involving *NR4A3*, *ZNF681*, and *EBF2*, which have a role in transcription regulation. Increased expression of NR4A3 on both protein and mRNA levels was found in HCC compared to PH [40], which we also detected on mRNA levels between HCC and PH, as well as HCC and HepG2. However, similar expression levels between PH and HepG2 indicate better usage of HepG2 to study hepatocytes for this specific gene. A similar observation was detected for PKLR, which is involved in glucose metabolism regulation, used in HepG2 cell lines to study both PH [41] and HCC [42], while our results suggest using it for studies as a model only for PH. The majority of 21 identified genes with similar expression in PH and HepG2 but with significantly differential expression in HCC are involved in the transmembrane transfer. Another contradictory usage similar to already described cases is that of gene *TRPC-6*. TRPC-6 is a Ca^2+^ permeant channel found to be overexpressed in HCC [43] but was investigated in HepG2 as a model cell line for HCC [44]. Overall, our findings claim that HepG2 is a good model for studies focused on fructose transportation through GLUT5, such as the ones by Hirahatake et al. [45] and Liang et al. [46]. However, the study by Huggett et al. shows that the effects of fructose differ in HepG2 compared to PH [47], indicating that the fructose pathways post-transportation into the cells differ in HepG2 and PH. The expression of *SIGLEC-15* was increased in HepG2 compared to the LO-2 human normal hepatic cell line and the HH primary human hepatocyte line on both protein and mRNA levels but was the lowest among six model HCC lines used in the study [48], indicating that HepG2 is the least ideal model for HCC in regard to this gene.

Among genes that our results show could reliably be researched in HepG2 as an HCC model, we found important genes involved in the DNA repair mechanisms. The genes whose expressions in HepG2 mirror their expression in HCC, according to our results, are ubiquitin-conjugating enzymes *UBE2T* and *PTTG1*. Multiple studies have observed a stimulating effect of UBE2T protein on the progression of HCC through the promotion of proliferation [49], cell migration [50], or epithelial–mesenchymal transition (EMT) [51], using HepG2 as a model. Fujii et al. found that *PTTG1* overexpression promotes angiogenesis in HCC and that its expression is an independent prognostic factor for disease-free survival (DFS) and overall survival (OS) [52]. Patients with higher expression of *RAD51AP1* have significantly worse OS and DFS than those with lower expression [53], which nicely implies the value of studying the *RAD51AP1* gene using HepG2 as a model for HCC. Similar findings were also found for OSBPL3 [54] and GNAZ [55]. Upregulation of the RAB11FIP4 protein product can lead to the increased metastatic potential of HCC [56], while upregulation of HKDC1, a member of the hexokinase protein family, is associated with the progression of HCC [57]. Multiple studies have demonstrated that IGF2BP1 and TRIM71 promote HCC progression in interaction with various types of RNAs (mRNAs or long non-coding RNAs (lncRNAs)) using HepG2 as a model cell line [58,59,60,61,62]. We did not find any studies on the remaining gene involved in DNA repair, the *HROB* gene, in HepG2, or in regards to HCC. The role of the *HROB* gene regarding HCC should be examined further in future studies.

We found that six genes involved in the regulation of immune response are similarly expressed in HepG2 and HCC. This finding can be viewed as a double-edged blade. On the one hand, HepG2 cells, being tumor cells, display gene expression patterns that correspond to those observed in HCC. However, it is important to note that HepG2 cells lack the microenvironmental cells present in tumor tissue, which are responsible for immune response regulation. Therefore, the expression of these immune response-related genes in HepG2 cells may not reflect their regulation in a tumor microenvironment. FNDC4, a protein involved in tumor growth factor β (TGFβ) response and downregulation of immune response, was found to be upregulated in HCC, and its upregulation was also associated with poorer overall survival of patients diagnosed with HCC [63]. The same study used HepG2 as a model cell line to display that upregulation of *FNDC4* promotes liver cancer cell migration and invasion in vitro via the PI3K/Akt signaling pathway [63]. *FGG* was shown to be overexpressed in both HCC and HepG2, matching our results, and increased levels of plasma fibrinogen correlated with the presence of tumor thrombosis and was associated with higher clinical stages of HCC [64]. The increased expression of KIF4A was found to be associated with poorer prognosis in HCC [65], while decreased expression of STC2 was associated with decreased cell proliferation and survival in HCC [66]. An example of a gene whose protein product has been explored as a target in HCC in multiple studies where HepG2 was used as a model is the cystine–glutamate transporter SLC7A11 [67,68].

Studying druggable genes presents a dual advantage: in HCC, it provides a framework for understanding cancer-specific vulnerabilities, while in PH, it helps elucidate the mechanisms of drug metabolism and liver-specific gene regulation. One instance is an inhibitor of serine proteases SERPINA1. *SERPINA 1* gene was identified as an important gene for the study of HCC using the HepG2 cell line. This gene is regulated by two drugs: igmesine and α 1-antitrypsin. It has been associated with chronic liver disease but has also been explored as a possible driver of HCC and other diseases using HepG2 as a model [69,70]. On the other hand, the druggable myeloperoxidase (*MPO*) gene is associated with the promotion of NASH-induced liver fibrosis [71], which later forms HCC. Exploring the modulation of these genes by specific drugs could lead to novel therapeutic strategies and improve our understanding of disease mechanisms in both cancerous and non-cancerous liver cells.

It is important to note that during the filtering process employed in our study, certain parameters were set to select genes. While we restricted these parameters to obtain the most confident results, it is crucial to acknowledge that the filtering process may still introduce some degree of arbitrariness. Also, this study could be further strengthened by performing cross-validation with additional datasets, particularly those representing precancerous states such as NAFLD, as well as incorporating data from HCC samples with various etiologies, given the heterogeneous nature of the cancer. Moreover, integrating multi-omics approaches—beyond transcriptome profiling, including epigenomics and genomics—could enhance the findings and potentially uncover new genes of interest. This could provide deeper insights when using HepG2 as a model for both PH and HCC research, making it a valuable avenue for future exploration.

## 5. Conclusions

Our study extensively investigated genes that could serve as reliable targets for exploring hepatocellular carcinoma and primary hepatocytes using the HepG2 cell line as a model. We compiled a comprehensive gene list specifically designed for HepG2-based studies, with a primary focus on HCC and hepatocyte research. We believe it is important to first identify which genes and pathways are most relevant for studying a disease before choosing cell lines as models. This way, researchers can save time and resources by focusing only on genes that are truly similar between patients with the disease and the cell line being used. By sharing this list of genes, we hope to give researchers a valuable tool to guide their studies and help uncover the underlying biology of liver cancer and liver cells using the HepG2 cell line as a model.

## Figures and Tables

**Table 1:** Summary of known functions of 21 and 40 DEGs for HepG2 as valuable models for primary hepatocytes (PH) and hepatocellular carcinoma (HCC).

Function	PH Model	HCC Model
Signal transduction	*KISS1*	*GNAZ*, *LRRC1*, *MYH4*, *STC2*, *DUSP9*
DNA repair		*RAD51AP1*, *HROB*, *UBE2T*, *PTTG1*
Protein degradation	*MAGEC2*, *DCAF4L2*	*UBE2T*, *DTL*, *MAGEA2*, *MAGEA2B*, *TRIM71*, *MAGEA6*, *SERPINA1*
Defense or immune response (inflammatory)		*DEFA1*, *DEFA1B*, *MPO*, *FNDC4*, *SIGLEC10*, *FGG*
Transcription regulation	*EBF2*, *NR4A3*, *ZNF681*, *MAGEC2*	*MAGEA2*, *MAGEA2B*, *MYBL2*, *MAGEA6*, *IGF2BP1*
Transmembrane transport (ion or/and molecule)	*GABRD*, *KCNJ4*, *TRPC6*, *SLC16A12*, *SLC2A5*	*CHRNE*, *KCNJ16*, *SLC13A5*, *SLC7A11*
Mitotic spindle regulation	*DYNC1I1*	*ASPM*, *GPSM2*, *KIF4A*, *MYBL2*, *NDC80*, *NUF2*, *PTTG1*
Neural communication	*PRIMA1*, *C1QL1*	
Other	*PKLR*, *ELFN2*, *C15orf48*, *NDUFA4L2*, *SIGLEC15*, *ERVFRD-1*, *C1orf210*	*SYN1*, *OSBPL3*, *PHACTR3*, *ACADS*, *SSUH2*, *RAB11FIP4*, *OIT3*, *HKDC1*

## Data Availability

All of the publicly available data has been described in detail in the Materials and Method Section. Sequencing of HepG2 cell lines is available upon request.

## Supplementary Materials

The following supporting information can be downloaded at: https://www.mdpi.com/article/10.3390/genes15111460/s1, Figure S1: Differential expression between hepatocellular carcinoma (HCC) and non-adjacent liver tissue, primary hepatocytes and non-adjacent liver tissue; Figure S2: Differentially expressed genes (DEGs) located in reference HepG2-specific karyotype regions; Table S1: Sample information; Table S2: Karyotype coordinates for HepG2; Table S3: Differential expression analysis results between pairwise groups; Table S4: Gene ontology (GO) over-representation analysis results; Table S5: KEGG (Kyoto Encyclopedia of Genes and Genomes) over-representation analysis results; Table S6: Differential expression analysis results compared to non-adjacent liver tissue; Table S7: Gene ontology (GO) over-representation analysis results of unique differentially expressed genes only between HepG2 and HCC, as well as HepG2 and primary hepatocytes; Table S8: The list of good potential genes for studying HepG2 as a representative model for primary hepatocytes and HCC.
